# Design and implementation of humidity sensor based on carbon nitride modified with graphene quantum dots

**DOI:** 10.1038/s41598-023-29960-8

**Published:** 2023-02-18

**Authors:** Mohamed Morsy, Islam Gomaa, M. M. Mokhtar, Hanan ElHaes, Medhat Ibrahim

**Affiliations:** 1 Building Physics and Environment Institute, Housing and Building National Research Center (HBRC), Dokki, Giza, 12311 Egypt; 2 Nanotechnology Research Center, The British University in Egypt (BUE), Suez Desert Road, El Sherouk City, Cairo, 11837 Egypt; 3 Physics Department, Faculty of Women for Arts, Science and Education, Ain Shams University, Cairo, 11757 Egypt; 4 Molecular Spectroscopy and Modeling Unit, Spectroscopy Department, National Research Centre, 35 El-Behouth St., Dokki, Giza, 12622 Egypt

**Keywords:** Materials science, Mathematics and computing

## Abstract

Relative humidity (RH) is one of the most important factors that deserve intensive study because of its impact on many aspects of life. In this work humidity sensor based on carbon nitride / graphene quantum dots (g-C3N4/GQDs) nanocomposites have been developed. The structure, morphology and composition properties of the g-C3N4/GQDs were investigated and analyzed by XRD, HR-TEM, FTIR, UV–Vis, Raman, XPS and BET surface area. The average particle size of GQDs was estimated from XRD to be 5 nm and confirmed using HRTEM. The HRTEM images prove that the GQDs are attached to the external surface of the g-C3N4. The measured BET surface area was found to be 216 m^2^/g, 313 m^2^/g, and 545 m^2^/g for GQDs, g-C3N4, and g-C3N4/GQDs respectively. The d-spacing and crystallite size were estimated from XRD and HRTEM and found in a good matching. The humidity sensing behavior of g-C3N4/GQDs was measured in a wide span of humidity from 7% up to 97% RH under different testing frequencies. The obtained results demonstrate good reversibility and fast response/recovery time. The implemented sensor exhibits a great application prospect in humidity alarm devices, automatic diaper alarms, and breath analysis, which have advantages such as strong anti-interference capability, low cost, and easy to use.

## Introduction

Relative humidity (RH) is one of the most important factors that deserve intensive study because of its impact on many aspects of life. The measurement and control of RH is one of the main objectives in many applications, like agriculture, pharmaceutical, electronic fabrication process, food industry, automotive and human-comfort zones^1–5^ For example, the thermal comfort for occupants within interior spaces of buildings depends basically on the RH level. The controlling and measuring the RH with acceptable accuracy within the interior spaces of buildings acquired noticeable importance^6,7^. Many strategies were employed to measure the level of RH, the most adequate one depends on tracing the change in one of physical properties of materials upon changing in RH level^8^. The basic criteria for humidity sensor are fast response, wide span of working range, good repeatability, long term stability as well as enhanced sensitivity^9,10^. Recently, several materials including semiconductor, ceramics^11–13^, conducting polymers, and carbon nanomaterials were explored as RH sensor^14^. Due to its distinctive electrical structure, graphitic carbon nitride (g-C_3_N_4_), a carbon compound that resembles graphite, has become a hot topic in materials science. One of the most promising photocatalytic materials has a medium band gap and is thermally and chemically stable in an ambient environment^15^. A stable allotrope among the others, g-C_3_N_4_ is made up of layers of 2-D polymeric structures with tri-s-triazine (C_6_N_7_) and s-triazine (C_3_N_3_) rings connected by tertiary amines^16^. Theoretical and experimental evidence points to the creation of delocalized bonds between the substituted carbon atoms and hexatomic rings when N atoms are replaced with C atoms. The electrical conductivity of g-C_3_N_4_ is increased by these delocalized bonds, which promote electron transport^17^. Additionally, the carbon self-doping contributes to the narrowing of the g-C_3_N_4_ bandgap, which enhances the absorption of visible light^18^. The scientific community is particularly interested in developing novel sensors with reliable characteristics. regarding its highly adaptable electrical and optical properties, high sensitivity and analyte selectivity, outstanding chemical stability, biocompatibility, high surface area, and inexpensive price, g-C_3_N_4_ is a brilliant option for sensing applications^16,18–20^.

Over decades carbon nano-based materials were investigated as an ideal candidate for humidity sensors. Graphene With the astonishing chemical and physical properties comes in the first order of sensor fabrication^9,21,22^. Carbon nanotubes (CNTs) have been extensively and intensively studied recently because of its distinct characteristics, which made it one of the distinguished materials in the applications of humidity sensors^23–25^. In comparison with carbon nano-based materials derivatives, graphene quantum dots (GQDs) have an elevated surface area, enrich in oxygen containing group, and more active sies which leading to promoting the humidity sensing performance^26^. Li et al.^27^ fabricate a capacitive humidity sensor based on GQDs/Nafion composite film for improving the sensitivity. Their experimental evidence demonstrated that the GQDs/Nafion sensor exhibits high sensitivity and better linearity than pure Nafion sensor. The *p*-type FET based on GQDs sensing layer was fabricated by ink-jet printing method and used as a transducer for humidity sensing application. Yujeong et al. found that the best performance of FET humidity sensor was recognized at 20 °C. Also, the response increases when + 2 V applied between control gate (CG), in contrary the recovery of the sensor enhanced when the − 1 V applied to CG^28^. Kondee et al. synthesized the nitrogen doped carbon quantum dots (NCQDs) via hydrothermal route. The humidity sensing characteristics of NCQDs elucidated a response of 90.97% in the range RH range 10–95%RH^29^. Pengjia et al. fabricate a quartz crystal microbalance (QCM) humidity sensor based on GQDs- chitosan. The fabricated sensor reveals a rapid response and recovery time of 36 s and 3 s @ 95% RH^30^. Molecular modeling is the applying quantum physics to study molecular structures. It includes several levels of theories such as semispherical; ab initio and density functional theory. It is performed via computer software to elucidate and obtain molecular data such as geometries, energies, electronic properties, spectroscopic properties etc.^31,32^. To investigate surface properties and functionality of a given structure one can calculate total dipole moment TDM; HOMO/LUMO band gap energy and mapping electrostatic potential (MESP)^33–35^.

The main target of this work is to attach a GQDs over g-C3N4 using ultrasonic. The structure of the resultant material verified through XRD, HR-TEM, FTIR, Raman, UV–Vis, BET and XPS. The humidity sensing properties of the obtained structure were tested in a wide span of humidity levels at different testing frequencies. The optimum testing frequency was affirmed based on the high impedance variation. The response time was increased as the GQDs alloyed with g- C3N4, while the recovery time enhanced. The recovery time for GQDs/g-C3N4 was found to be 10 sce. The sensing mechanism were explained using complex impedance spectroscopy measurements. Additionally, DFT at B3LYP/LANL2DZ is conducted to calculate TDM; HOMO/LUMO band gap and MESP for g- C3N4, GQDs as well as their composite. The obtained results are encouraged as the sensitivity of the g-C3N4 was enhanced as it alloyed with GQDs.

## Experimental

### Chemicals

Citric acid (99.5%), urea (97%) and sodium hydroxide (97%) were supplied from the fisher. The deionized Milli-Q water was used during this experiment.

### Materials synthesizing

#### Graphene Quantum Dots (GQDs)

Graphene Quantum Dots (GQDs) have been prepared using citric acid as a carbon source. Briefly 5 g of citric acid was melted at 180 °C for 12 h, 1.5 M solution of NaOH was added drop wise to the melted dense solution of citric acid at room temperature, finally hydrothermal treated at 180 °C for additional 12 h. The resultant dark brown powder was separated from liquor via dialysis filtration, washed and dried in vacuum oven at 70 °C overnight.

#### Carbon nitride (g-C3N4)

The g-C3N4 was obtained through a well-known calcination method using Urea as precursors. In brief 10 g of urea was grounded in a ceramic mortar for 10 min, then placed in covered alumina crucible and heated from room temperature up to 550 °C in air with heating rate of 5 °C min^−1^. The sample was held at 550 °C for 3 h, subsequently and after cooling naturally, the obtained yellow flakes were collected and grounded into a fine powder.

#### GQDs\g-C3N4 nanocomposite

**Figure 1:**
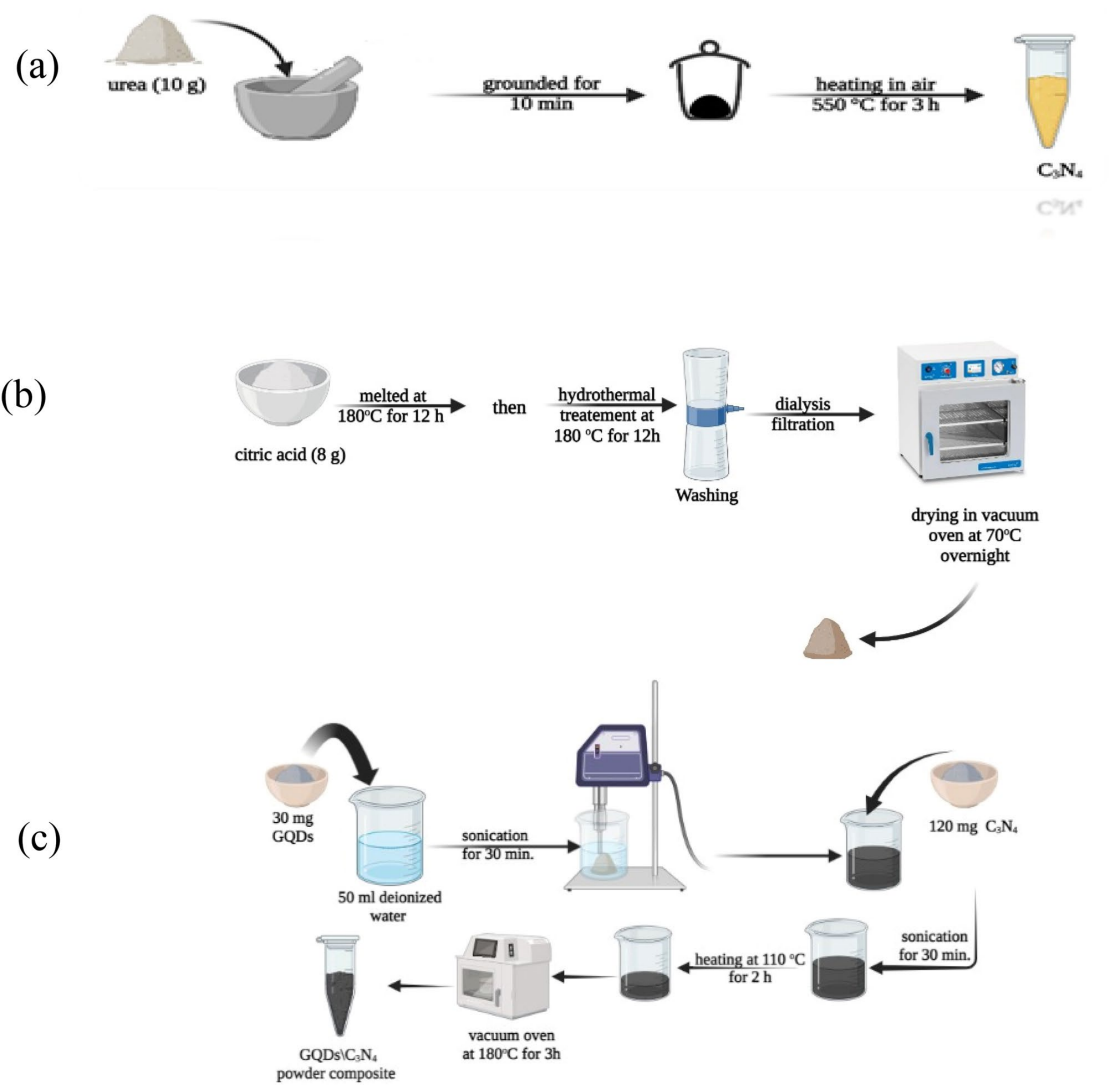
Schematic representations of materials synthesizing procedures (**a**) g C3N4, (**b**) GQDs, and (**c**) GQDs/g-C3N4 composite.

The GQDs\g-C3N4 nanocomposite was prepared using ultrasonication method. 30 mg of GQDs was dispersed in 50 ml of deionized water for 30 min using ultrasonic probe sonicator. The probe sonicator was operated repeatedly in on–off mode with 10 s cycles. After confirming the good dispersion of GQDs, 120 mg of g-C3N4 was added to the GQDs suspension and subjected to sonication for additional 30 min. The water evaporated through two successive processes. Eventually, the first comprises heating at 110 °C for 2 h, then transferred to vacuum oven at 180 °C for 3 h. The mixing percent of GQDs to g-C3N4 is 20% as the weight ratio The obtained composite in addition to its components were subjected to detailed investigation as will be explained herein after. The schematic diagram of all experimental details for GQDs, g-C3N4, and GQDs/g-C3N4 is represented schematically in Fig. 1a, b, and c.

### Calculations details

All the studied structures were calculated using Gaussian 09^36^ [6] at Molecular Modeling and Spectroscopy unit, Spectroscopy Department, National Research Centre. The studied model molecules were calculated at density functional theory B3LYP^37–39^ with LANL2DZ basis set. Some important physical parameters were calculated at the same level of theory namely, total dipole moment (TDM), the highest occupied molecular orbital (HOMO) and a lowest unoccupied molecular orbital (LUMO), and Molecular electrostatic potential (MESP).

### Characterization techniques

The composition, phase structure, and physical and chemical properties of the prepared structures were verified using different characterization techniques. The phase structure and composition were verified by XRD measurements. The XRD patterns were recorded using Malvern Panalytical Empyrean 3 diffractometer. The microstructure, and morphology of the synthesized materials were investigated using HRTEM model JEM 2100, JEOL, Japan. The attached functional group and possible interaction between different constituents of materials were verified throughout FTIR measurements. The FTIR spectra were acquired in a spectral range of 4000–400 cm^−1^ using FTIR spectrometer (Vertex 70, Bruker). The UV–Vis spectra of all prepared materials were acquired by spectrophotometer (Jasco V-570, Spain) in a spectral range of 200–800 cm^−1^. The electronic band structure was verified UV–Vis spectrophotometer model Agilent Cary5000. The Raman spectra of the samples were measured using a confocal Raman microscope (Witec Alpha 300 RA, 514 nm excitation). The N2 Brunauer–Emmett–Teller (BET) surface area was measured by means of Quantachrome NOVA 2200E apparatus. The X-ray photoluminescent spectroscopy (XPS) spectra were acquired by K Alpha instrument (Thermo Fischer, Al Kα radiation) to confirm the elemental composition and oxidation state.

### Sensor fabrications and testing

**Figure 2:**
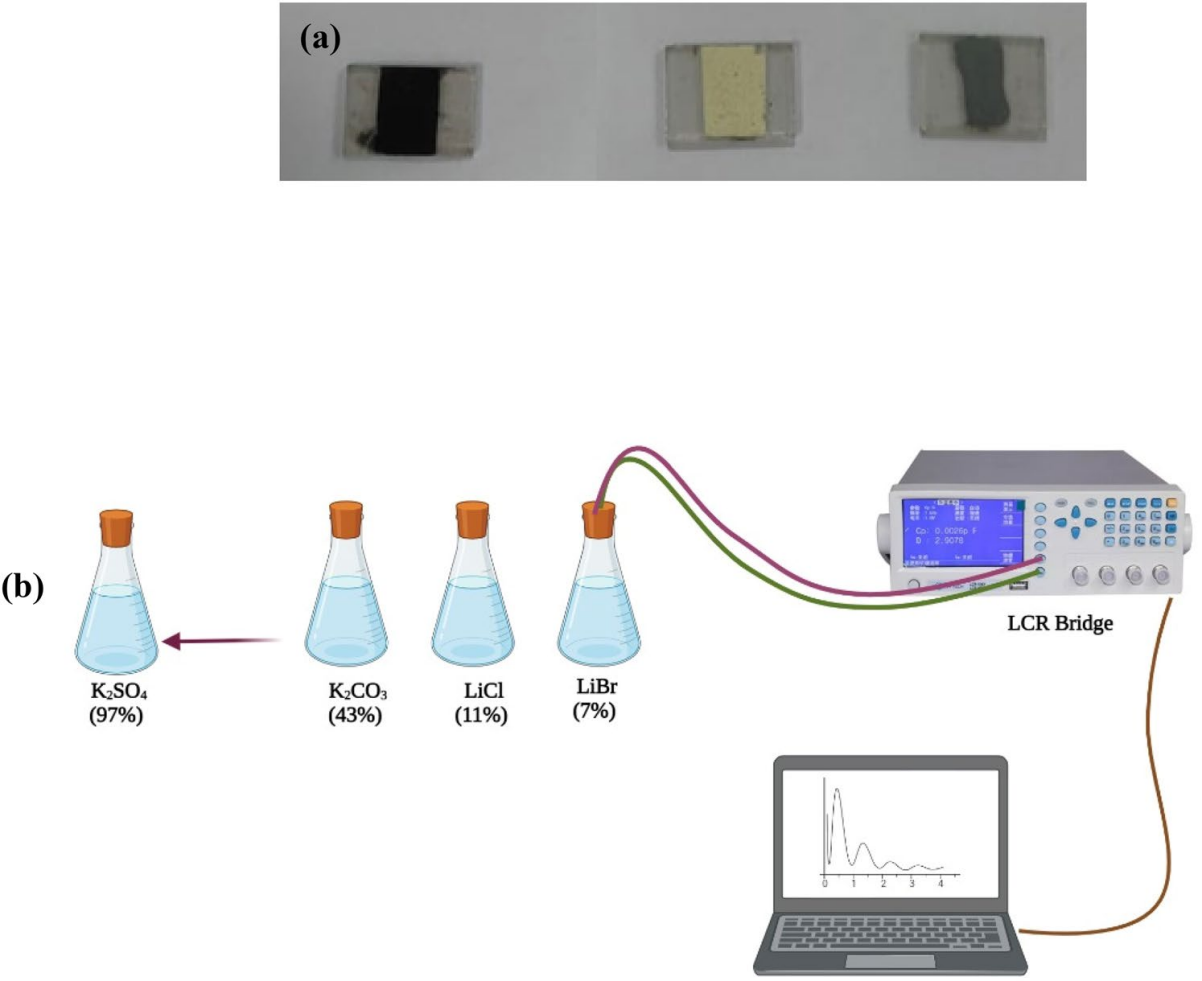
(**a**) The photograph of humidity sensor and (**b**) The humidity sensing testing setup using saturated salt solutions.

The measured sensors were cast over florinated tin oxide (FTO) coated glass substrate. In brief a minimal amount of the sensing materials was mixed gently with a surplus amount water to form past, then spread over a substrate using spin coating technique. The fabricated sensors allowed to dry over-night at 60 °C then allowed to age under 1VAC for 24 h. The humidity sensing experiment was carried out using saturated salt solutions in a closed flask. The sensors were evaluated in a wide range of humidity (7% up to 97%) under different testing frequencies from 50 Hz to 100 kHz using LCR meter (Hioki-50). More information regarding the testing condition and sensor fabrication is explained in previous reported articles^5,21,40,41^ All humidity sensing experiments were performed at room temperature. The photograph of the prepared sensor (1.3 cm × 2 cm) is represented in Fig. 2a while, the testing experiment setup is represented in Fig. 2b

.

## Results and discussion

**Figure 3:**
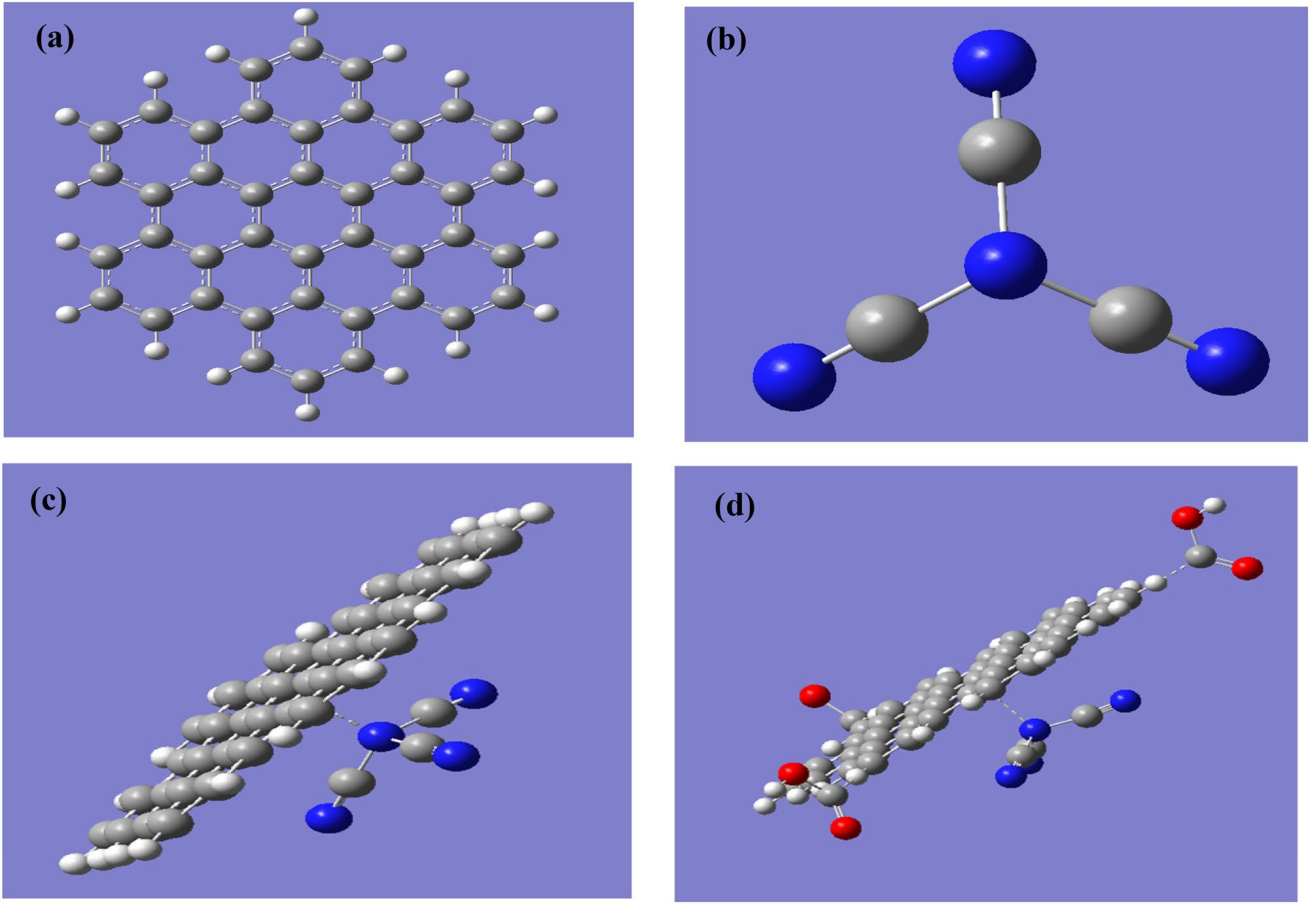
Optimized model molecules (**a**) Graphene quantum dots GQDs; (**b**) C3N4; (**c**) C3N4/GQDs and (**d**) C3N4/GQDs.3COOH.

As indicated in Fig. 3 four model molecules were built. Figure 3a presents the hexagonal graphene quantum dots (GQDs) with armchair termination which is termed AHEX. Figure 3b presented carbon nitride (g-C3N4). Figure 3c graphene quantum dots which is overlaped over g-C3N4 forming GQDs/g-C3N4. Figure 3d presented the model for GQDs/g-C3N4with 3 carboxyl group interacted with GQDs forming GQDs/g-C3N4.3COOH. The studied four model molecules were calculated with B3LYP/LANL2DZ level.

**Figure 4:**
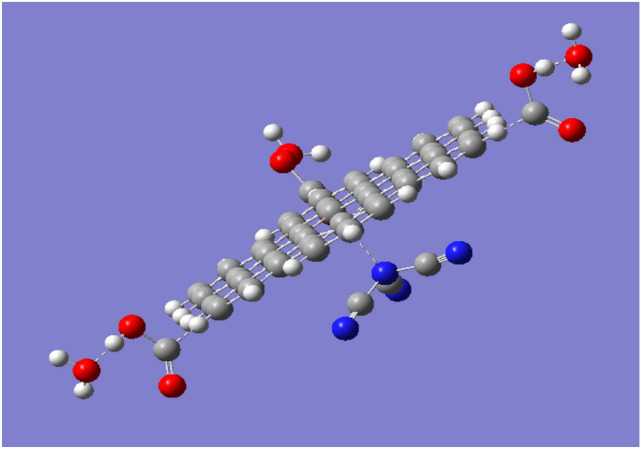
Optimized GQDs.g-C3N4. 3COOH which interacted with 3 water molecules.

A model which described the possible interaction between g-C3N4/GQDs.3COOH and humidity is indicated in Fig. 4. As shown in Fig. 4, three water molecules interacted with g-C3N4/GQDs.3COOH through carboxyl groups. The model indicated that oxygen of each water molecule interacts with hydrogen of the carboxyl group with weak (Van der Waal interaction).

**Figure 5:**
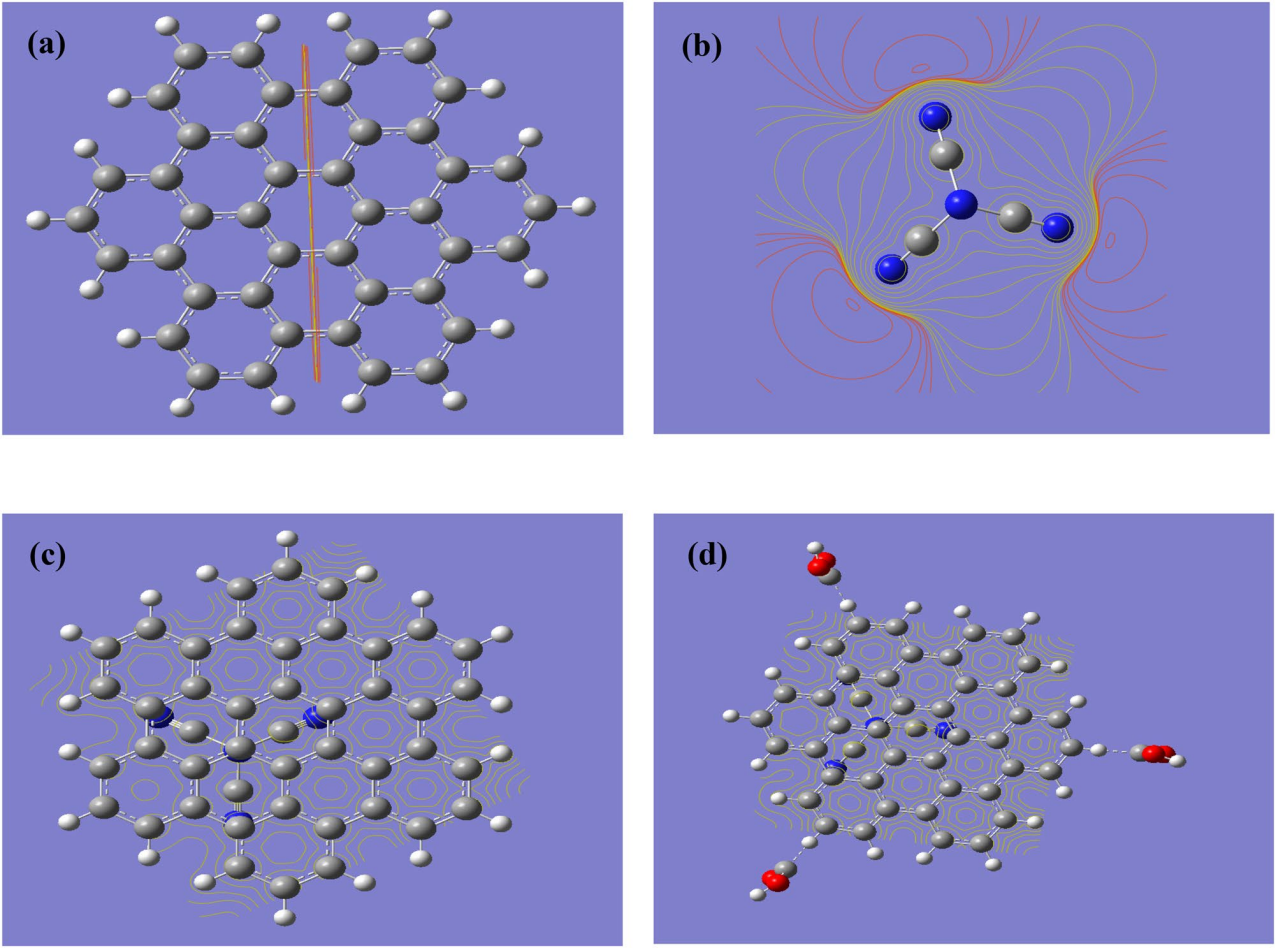
Mapping molecular electrostatic potential at B3LYP/LANL2DZ for the (**a**) Graphene quantum dots GQDs; (**b**) g-C3N4; (**c**) C3N4 /GQDs and (**d**) C3N4/GQDs.3COOH.

Figure 5 presented the mapping of the molecular electrostatic potential MESP at B3LYP/LANL2DZ for the GQDs as indicated in Fig. 5a; the g-C3N4 as indicated in Fig. 5b; the GQDs overlaid onto carbon nitride as indicated in Fig. 5c and finally GQDs overlaid onto carbon nitride functionalized with 3 carboxyl groups as shown in Fig. 5d.

MESP describes the reactivity of the given structure in terms the color scheme, whereas red corresponding to negative to blue indicating positive sites^42^. The negativity decreases on going from red, yellow, green, to blue. It is an indication for the active sites in a given surface. Applying this to the surface of GQDs.

As stated in Table 1 the total dipole moment for GQDs was 0.000 Debye while its band gap energy was 3.5084 eV then the same valued for g-C3N4 were 1.2053 Debye and 7.5640 eV respectively. As GQDs overlaid onto g-C3N4 the TDM increased up to 1.9445 Debye while the band gap decreased to be 3.3269 eV. For the functionalized g-C3N4/GQDs with carboxyl group the table indicated that the existence of carboxyl increases the TDM to be 5.5136 Debye while the band gap energy is decreased to be 2.1220 eV.

**Table 1:** B3LYP/LANL2DZ calculated total dipole moment TDM as Debye, HOMO/LUMO band gap energy as eV for the studied model molecules.

Structure	Physical parameters	
	TDM	HOMO/LUMO
GQDs	0.0000	3.5084
g-C3N4	1.2053	7.5640
g-C3N4/GQDs	1.9445	3.3269
g-C3N4/GQDs.3COOH	5.5136	2.1220
g-C3N4/GQDs.3COOH.3H_2_O	8.9571	2.8216

**Figure 6:**
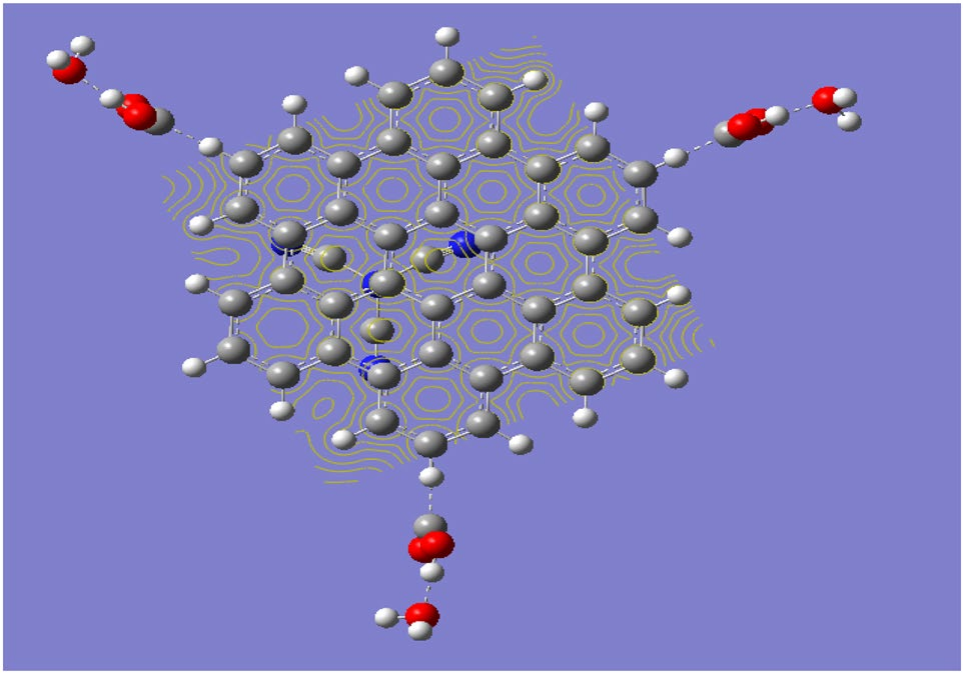
Mapping molecular electrostatic potential at B3LYP/LANL2DZ for the g-C3N4/GQDs.3COOH after interaction with 3 water molecules.

Correlating both results one can conclude that, QGDs over g-C3N4 show uniform MESP with reactive surface in terms higher TDM with lower band gap energy. It is stated earlier that both dipole moment and band gap energy are good descriptors for the reactivity of a given chemical structure^43,44^. This may dedicate the studied surface as sensor. Another model for possible interaction between GQDs.g-C3N4 0.3COOH and three water molecules are indicated as in Fig. 5d, its MESP is indicated in Fig. 6

.

The calculated TDM was increased as 8.9571 Debye while the band gap energy is 2.8216 eV. These data revealed that the g-C3N4/GQDs.3COOH could be a good substrate for interaction with humidity through adsorb state (Van der Waal interaction) and subsequently it can be dedicated for humidity sensor, which is in good agreement with the previous findings for utilizing carbon-based materials as sensor^45^.

Such modeling approaches need verifications, the first is to conduct experimental verifications in order to prepare; characterize g-C3N4/GQDs.3COOH then, performing humidity sensor experiment. These will be conducted as in the following parts.

**Figure 7:**
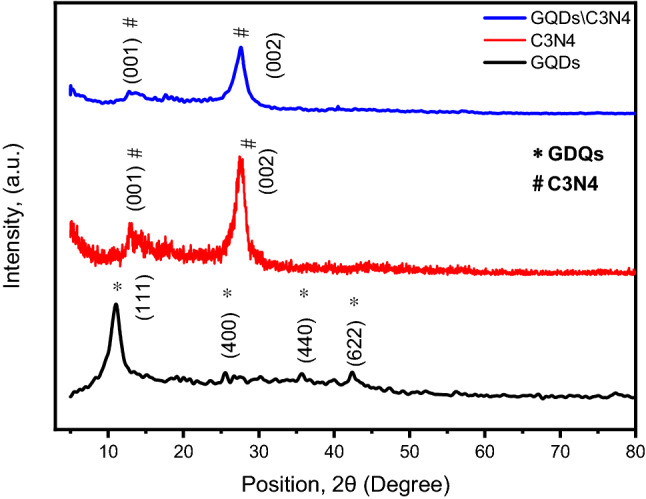
The XRD patterns of GQDs, g-C3N4 and GQDs\C3N4.

The phase identifications of the prepared composites were verified by XRD measurements. The diffraction patterns of the GQDs, g-C3N4, and GQDs/g-C3N4 were investigated in Fig. 7. The XRD pattern of GQDs demonstrate diffraction peaks at 11.05°, 25.57°, 35.65°, and 42.33° that can be indexed to the (111), (400), (440), and (622) which coincidence with card No. 01-082-2261. For XRD pattern of g-C3N4, the diffraction peaks at 12.9°, and 27.5° is indexed to the diffraction planes of (001) and (0 0 2) respectively^46^. The XRD pattern of the GQDs/g-C3N4 represents peak at 12.75° that can be ascribed to (111) diffraction plane of GQDs. While the peak at 27.4° is attributed to the (002) diffraction plane of g-C3N4. It can be noticed that the main diffraction peak of GQDs was absent. This could be due to the amount of GQDs is relatively low when compared to the g-C3N4 and incorporation of the GQDs between g-C3N4 layers. The purity of the prepared materials has been confirmed due to the absence of the diffraction peaks arose from any impurities. The mean crystallite size of the GQDs was estimated based on the well-known Deby Scherr equation as represented in Eq. (1). The dislocation density (δ) and micro-strain (ε) were calculated using the Eqs. (2) and (3)^47^.

1 $$D \left( {crystallite size} \right) in nm = \left[ {\frac{\left( k \right) \times \left( \lambda \right)}{{\left( {\beta_{D} } \right) \times \left( {Cos\theta } \right)}}} \right]$$

2 $$\delta = {\raise0.7ex\hbox{$1$} \!\mathord{\left/ {\vphantom {1 {D^{2} }}}\right.\kern-0pt} \!\lower0.7ex\hbox{${D^{2} }$}}$$

3 $$\varepsilon = \beta cos\theta \backslash 4$$

The crystallite size was estimated using the full width at half maximum (FWHM) of the most intense peak. The calculated particle size for GQDs was estimated and found to be 5 nm. The d-spacing of the prepared materials are as follows 8.06 A°, 3.244 A°, and 3.25 A° for GQDs, g-C3N4, and GQDs/g-C3N4 respectively. The d-spacing values of the GQDs/g-C3N4 has been slightly changed due to the incorporation of GQDs over g-C3N4 sheets. All the estimated parameters are visualized in Table 2.

**Table 2:** The crystal structure parameters of GQDs, C3N4, and GQDs/g-C3N4.

Sample name	(hkl)	*d-spacing*	*β*_D_ (°)	*D* (Scherrer eq.) (nm)	*δ* (nm^2^)	ε
GQDs	(111)	8.06	0.0268	5.2	0.037221	0.069432
g-C3N4	(002)	3.24	0.0283	*–*	0.039455	0.028885
GQDs/C3N4	(002)	3.25	0.0445	*–*	0.084571	0.023732

The dislocation densities of the prepared materials were increased as the incorporation of the GQDs into g-C3N4 matrix. The dislocation density implies introducing a crystallographic defect in the microstructure of the materials. The presence of dislocations affects the different properties of the studied materials. For example, the hardness of the materials increases as the number of dislocations increases^47,48^. Not only that, but dislocations also act as active sites for enhancing the sensitivity of the materials toward different stimuli. In contrary, the micro strain decreases.

**Figure 8:**
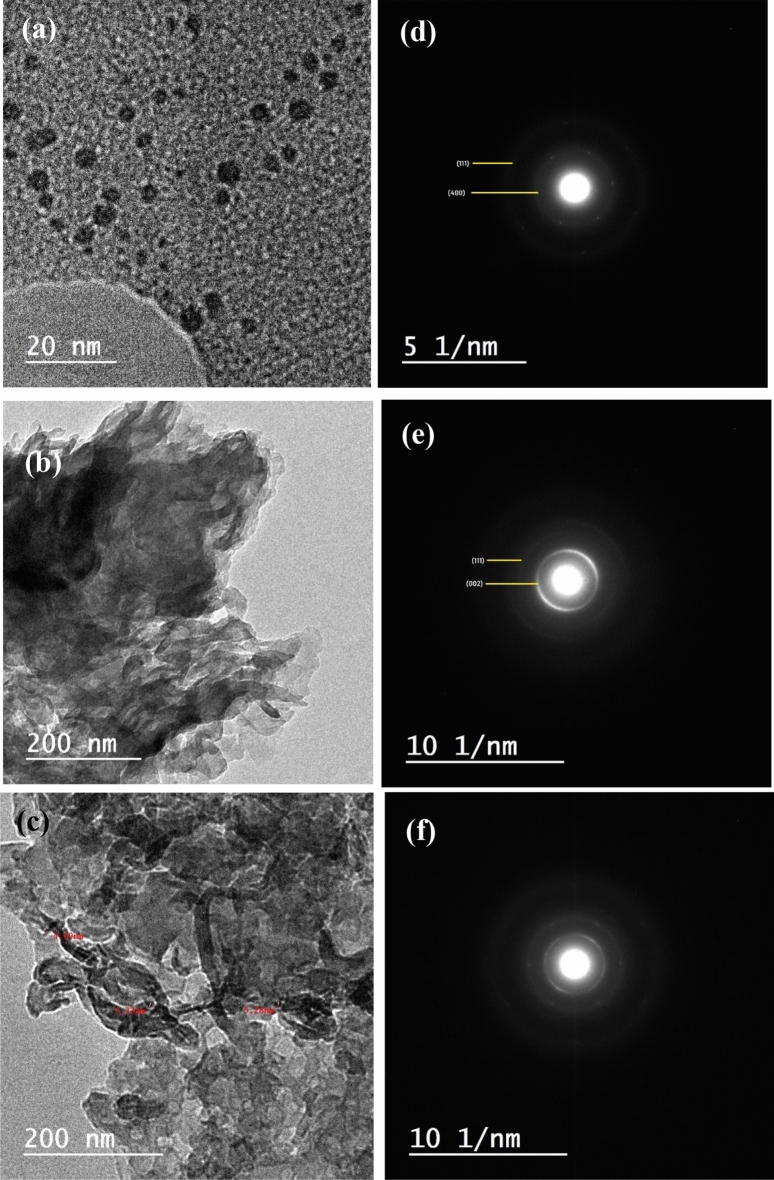
The HRTEM with corresponding SAED of (**a**) GQDs, (**b**) g-C3N4, and (**c**) GQDs/g-C3N4, (**d**) SAED of GQDs, (**e**) SAED of g-C3N4, and (**f**) SAED of GQDs/g-C3N4.

The detailed microstructure of the prepared samples has been investigated using the HRTEM, where the morphological features, crystallite size, and selected area electron diffraction (SAED) are visualized in Fig. 8. The samples were suspended in DMF and dispersed using ultrasonic waves for enough time to ensure the good dispersion, then loaded over a carbon coated gride. The contrast and stains in the background may be due to the DMF. The HRTEM of GQDs (Fig. 8a) demonstrates well dispersed spherical particles with average dimension of about 4 nm. The measured paticle size from HRTEM is compatible with that estimated size from XRD pattern. For the g-C3N4, the morphological features of Fig. 8b seem to be 2D wrinkled overlapped sheets with irregular shape. The 2D sheets seem to be fluffy and porous. Through this work, the 2D structure of the g-C3N4 was used as a scaffold for loading GQDs. As can be seen from the HRTEM of the GQDs\g-C3N4 sample represented in Fig. 8c, the GQDs having spherical shape are attached to the surface of g-C3N4. The HRTEM images demonstrate the successful loading GQDs over the 2D g-C3N4.

The SAED of the samples exhibited a poly crystalline structure. For the GQDs, the carbon atoms are clearly visualized as 6 bright spots as shown in Fig. 8d. The concentrated continuous rings appear in the SAED of g-C3N4 sample proves the polycrystalline structure of the sample as shown in Fig. 8e. Meanwhile the SAED of the GQDs\g-C3N4 composite (Fig. 8f) exhibited bright spots in addition to continuous rings confirming the presence of GQDs and g-C3N4 in the examined sample. The estimated d-spacing values as derived from XRD and SAED is comparable as seen in Table 3.

**Table 3:** The d-spacing estimated from XRD and SAED.

Material	(hkl)	Position, 2θ	d-spacing, XRD	d-spacing, HR-TEM
GQDs	(111)	11.0	8.06	8.01
g-C3N4	(002)	27.5	3.24	3.31
GQDs\g-C3N4	(111), (002)	11.05, 27.5	8.06, 3.24	8.25, 3.28

**Figure 9:**
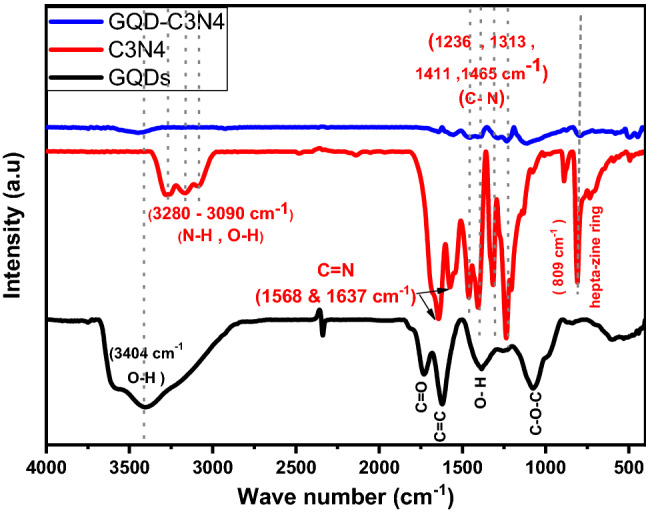
The FTIR spectrum of the GQDs, g-C3N4, and GQDs/g-C3N4.

The FTIR spectra measured in spectral range from 4500 to 400 cm^−1^ is directed in Fig. 9. The FTIR spectra of GQDs can be easily differentiated from those of pure GO. The stretching vibrations of –OH, C=O, C=C, and C–O–C are represented by the peaks at 3404 cm^−1^, 1731 cm^−1^, 1615 cm^−1^, and 1077 cm^−1^, respectively^49,50^. In g-C3N4 and composite. The FTIR chart show Peaks at 808 cm^−1^ correspond to the finger print vibrational energy pattern of the g-C3N4 of heptazine structure, whereas three distinct peaks  in spectral range 3280–3090 cm^−1^ correspond to the stretching vibration of N–H/O–H, and several strong absorption peaks in the 1236–1465 cm^−1^ region are associated with the stretching vibration of the C-N heterocycle^19^. The two sequenced peaks at 1568 and 1637 cm^−1^ regard to C=N heterocycles. Due to non-destructive loading via ultra -sonication and uniform distribution of GQDs, the FITR spectra of g-C3N4 and GQDs did not exhibit any discernible differences, suggesting that creation of the GQDs/g-C3N4 hetero structures did not damage the conjugated CN structure of the C_3_N.The FT-IR spectra exhibited overlapping interactions of N–H, CH and OH in GQDs and g-C3N4 to sharp peak at 3451 cm^−1^ in Composite form^18^.

**Figure 10:**
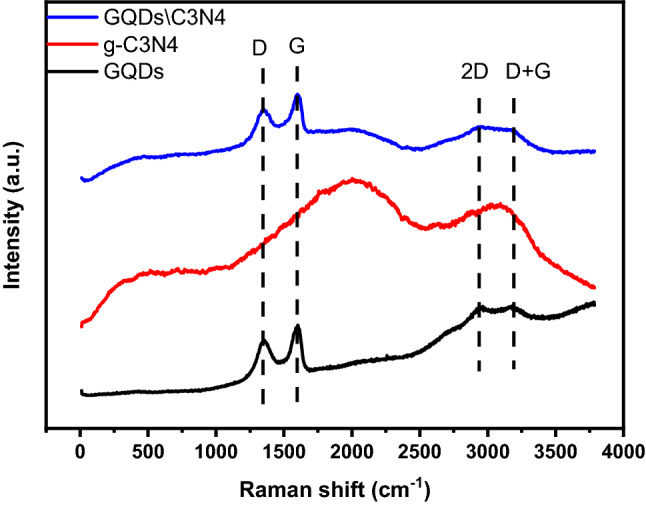
The Raman spectrum of the prepared materials.

Figure 10 exhibits the Raman spectrum of the prepared materials. The g-C3N4 sample exhibits two broad bands centered around 2000 cm^−1^ and 3090 cm^−1^. For GQDs, two prominent bands can be recognized, in addition to two broad bands. The D band at 1340 cm^−1^ is due to the disordered originated from SP^2^ hybridized carbon, while the G band at 1595 cm^−1^ associated with the first order scattering of the stretching vibration mode E_2g_ remarked for sp^2^carbon domains. The 2D and D + G broad bands centered around 2943 cm^−1^ and 2512 cm^−1^ originated due to relaxation in the selection rules formed by phonon scattering at boundaries and defects in the GQDs^51,52^. The values intensity ratio of the D band to G band (I_D_\I_G_) of GQDs and GQDs\C3N4 samples were 0.9 and 0.95. The I_D_\I_G_ is a structural metric related to the defect density and crystallite size. It can be concluded that the defect density increased due to the combination between g-C3N4 and GQDs. The average cluster size was obtained from the I_D_\I_G_ ratio of Raman spectra using the Tuinstra and Koenig (TK) equation^52^:

4 $$\frac{{I_{D} }}{{I_{G} }} = C\left( \lambda \right) L^{2}$$

where C(λ) is a constant depends on the excitation laser wavelength and L is the cluster size.

The estimated cluster size was found to be 1.3 nm. Comparing this value with that obtained from Scherr equation a difference in size is noticed. However, the HRTEM demonstrates the presence of particles in the range of 1–2 nm. Concluding the above results leading to say that the average size is 2–5 nm.

**Figure 11:**
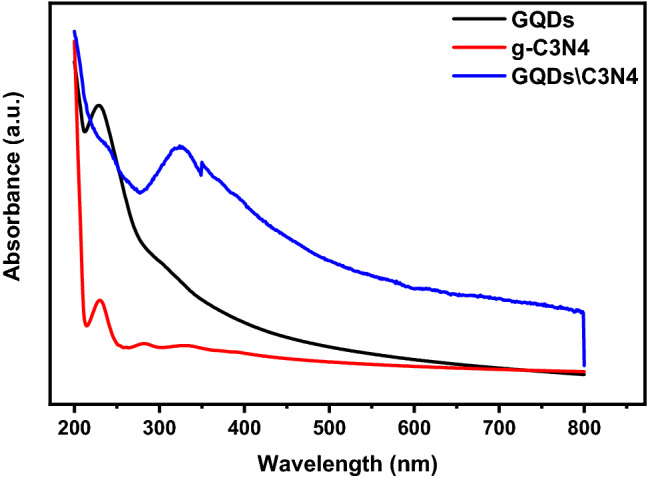
The UV–Vis absorbance of prepared materials.

The electronic band structure of the prepared materials has been studied using UV–Vis spectrophotometer from 200 up to 800 cm^−1^ as shown in Fig. 11. The UV–Vis spectrum of the GQDs reveals an absorption band at 230 nm which can be assigned to the π-π* transition of aromatic C=C sp2 domains^53^. The absorption bands at 200–350 nm of g-C3N4 is originated due to transferring charges from valence band (VB) of nitrogen atom to a conduction band (CB) of carbon atom of carbon nitride. The band tailing in the range from 350 to 500 nm indicates a slight visible light absorption of g-C3N4^47,54^. The UV–Vis spectrum of GQDs\C3N4 composite demonstrate absorption bands at 230 nm due to the π-π* transition of aromatic C=C sp2. While bands at 320 nm originated from the charge transferring from VB to CB.

**Figure 12:**
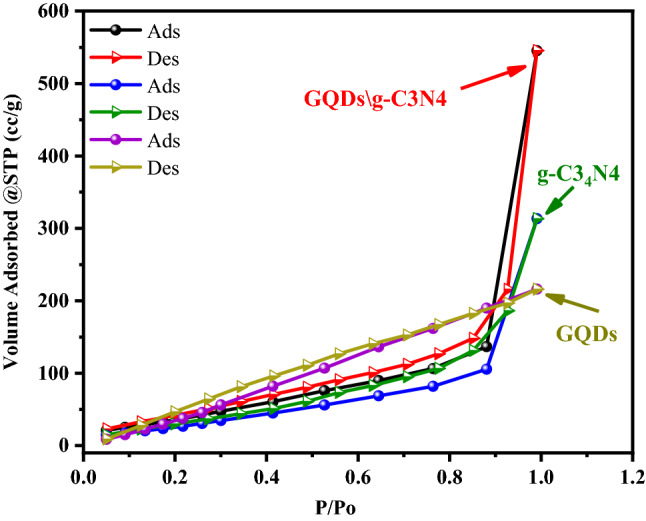
The N_2_ adsorption/desorption isotherms of the prepared samples.

The specific surface area and pore volume of the GQDs, g- C3N4, and GQDs\g-C3N4 samples were measured using the N_2_ adsorption/desorption isotherms presented in Fig. 12.

According to the International Union of Pure and Applied Chemistry (IUPAC), the N2 adsorption–desorption isotherm of GQDs, g-C3N4, and GQDs\g-C3N4 are of type III^55,56^. The estimated BET surface area was 216, 313.4, and 545.4 m^2^/g for GQDs, g-C3N4, and GQDs\g-C3N4 respectively. The highest surface area was observed for GQDs\g-C3N4 as a result of successful combination between g-C3N4 and GQDs. The elevated BET surface area could be due to the incorporation of GQDs between g-C3N4 sheets that preventing the agglomeration, thereby increasing the surface area^21^. This assumption is confirmed by HRTEM where the GQDs were observed as dark dots impeded between the g-C3N4 nano sheets.

The estimated BJH surface area, pore volume and pore radius for all prepared samples is presented in Table 4.

**Table 4:** The parameters of the complete N2 adsorption–desorption isotherm of all samples.

Sample name	Average Pore Size, nm	BET surface area, m^2^/g	BJH surface area, m^2^/g	BJH Pore Volume, cm^3^/g	Total Pore Volume
GQDs	1.9159	216	175.164	0.297793	0.33506
g-C3N4	8.2217	313.4	88.2405	0.462405	0.48604
GQDs/g-C3N4	1.0654	545.4	112.742	0.810872	0.84573

**Figure 13:**
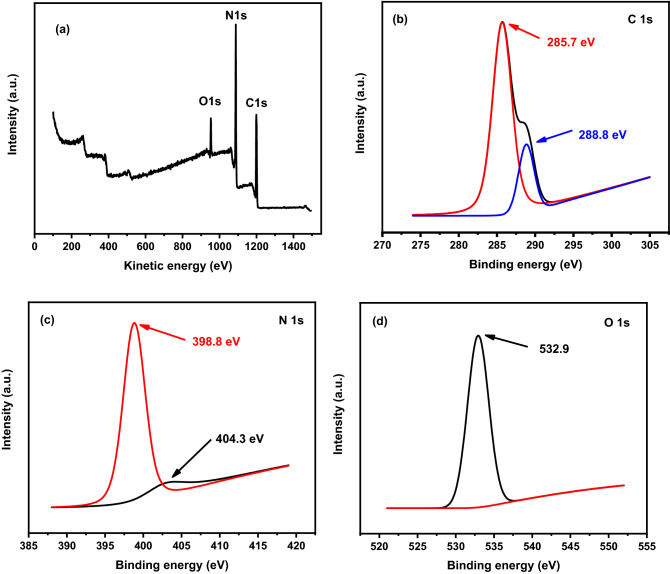
XPS spectrum of the GQDs\g-C3N4 sample: (**a**) Survey, (**b**) C 1 s, (**c**) N 1 s and (**d**) O 1 s.

The X-ray photoelectron spectroscopy (XPS) analysis is a helpful and powerful technique used mainly to specify the constituent elements of a material and oxidation states. The surface elemental composition analysis has been performed for the GQDs\g-C3N4 sample using XPS in the spectra range 0–1400 eV as shown in Fig. 13. The measured XPS survey scan spectrum shown in Fig. 13a is characterized by three very sharp peaks at 953 eV, 1088 eV, and 1199 eV corresponding to O 1 s, N 1 s, C 1 s respectively. The spectrum of the C 1 s shown in Fig. 13b was deconvoluted to two peaks at 285.7 eV, 288.8 eV that assigned to SP^2^ of C–O/C–O–C, and O=C–O/N–C=N bonding respectively^57^. The carbon atom on the aromatic ring connected to NH_x_, and the sp^2^ hybrid carbon atom connected to the nitrogen atom in the triazine ring N–C=N^58^. For the results of N 1 s peak deconvolution represented in Fig. 13c, the two peaks at 398.8 eV and 404.3 eV is arising from the C–N=C and N–O respectively^59^. The high-resolution spectrum of Fig. 13d exhibited single peak at 532.9 eV corresponding to C–O.

### Humidity sensor measurements

**Figure 14:**
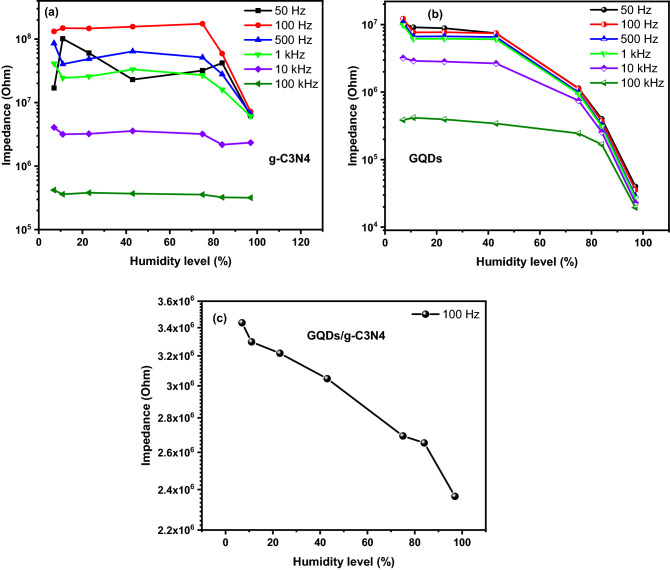
The humidity sensing characteristics of the (**a**) g-C3N4 at different frequencies, (**b**) GQDs at different frequencies, and (**c**) GQDs/g-C3N4 at 100 Hz.

The humidity response at different frequencies for all prepared samples were measured to determine the optimum testing frequency. The samples were tested in a frequency range from 50 Hz up to 100 kHz. The samples were conditioned at 7% RH and 97% RH for 24 h to enhance sensor response and reduce signal to noise ratio. The maximum variation of g-C3N4 sensor was affirmed at 100 Hz for humidity levels more than 75% as depicted in Fig. 14a. This behavior could be due to the lack of hydroxyl group attached to the external surface of g-C3N4. Referring to the obtained results, the g-C3N4 sample will not be further considered as a humidity sensor. As can be seen from Fig. 14b, the impedance values of GQDs decrease as the humidity increases from 7 to 97%. The impedance starts to decrease slightly up to 43%, then rapidly decreases in a linear manner.

As confirmed earlier, the maximum impedance variation was achieved at 100 Hz. The subsequent evaluation of the sensor will be conducted at 100 Hz.

The GQDs exhibits a distinct behavior compared to g-C3N4 when subjected to different levels of humidity. The impedance variation curves as a function of humidity level at different testing frequencies are displayed in Fig. 14. For all testing frequencies the impedance decreases with further humidity increasing. This is the expected trend for all humidity sensors that are related to the adsorption of water molecules on the surface of the sensing material. It is worth mentioning that the best response was achieved at 100 Hz. The optimum testing frequency is related to the ability of adsorbed water molecule to polarize at specific frequency. As the frequency increases the water molecule cannot follow the polar alternation, thereby a minor change in the impedance values is attained^3,5,21,60^. Further evaluation for humidity sensor were performed at 100 Hz. The humidity response of GQDs/g-C3N4 composite at 100 Hz displayed in Fig. 14c demonstrates a linear dependance of impedance as humidity increases. The normalized sensitivity, hysteresis and response and recovery time represent the most valuable parameters that deserve more investigation. The sensitivity and hysteresis can be estimated using the following equations^4^:

5 $$S = \frac{{Z_{7 } - Z_{H} }}{{RH_{H } - RH_{7} }}$$

6 $$H = \frac{{Z_{D} - Z_{A} }}{S}$$

**Figure 15:**
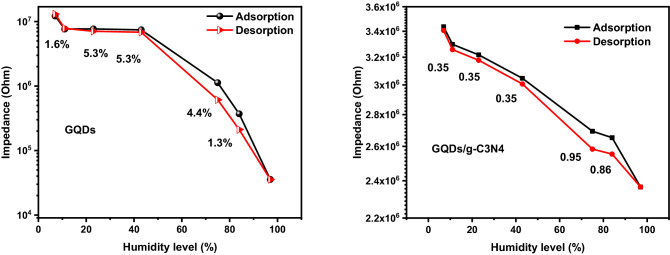
The hysteresis of the prepared composites (**a**) GQDS, and (**b**) GQDs/g-C3N4.

The GQDs sensor demonstrate sensitivity of 0.1 MΩ\RH. For the GQDs/g-C3N4 nanocomposite the compared to GQDs/g-C3N4, but the linearity of the GQDs/g-C3N4 encourage it to be the best candidate for humidity. As can be seen from Fig. 15, the hysteresis of the GQDs/g-C3N4 shows a small values (less than 1) at entire testing range compared to GQDs. This important parameter (hysteresis) confirms that the composite material has a key feature represented in linearity and less hysteresis over its individual constituents.

**Figure 16:**
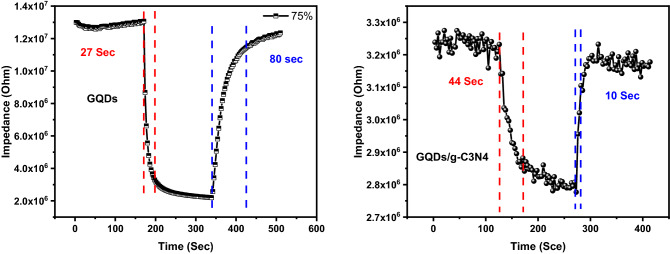
The response and recovery time of GQDs and GQDs/g-C3N4.

The ability of humidity sensor to respond to humidity in a short period of time is an added value. The evaluation of both response and recovery time for GQDs and GQDs/g-C3N4 is represented in Fig. 16. The sensors were subjected to a lower level of humidity 7% and high level of humidity 75% for 5 min. The response time for GQDs and GQDs/g-C3N4 was 27 s and 44 s respectively. The recovery time for GQDs and GQDs/g-C3N4 was estimated and found to be 80 s and 10 s respectively. It can be concluded that the response time increased as the GQDs overlapped g-C3N4 while the recovery time decreased. This behavior could be explained by following the active sites that adsorb water molecule. For the composite material a little amount of GQDs is loaded over the 2D g-C3N4 nano sheets. It was confirmed previously that GQDs reach of functional group that act as an active site for water molecule adsorption. When the amount of GQDs loaded over 2D g-C3N4 decreases, the amount of active sites decreases, thereby less water molecules are adsorbed, hence the sensor takes a prolonged time to respond to humidity. For the recovery time, as the amount of adsorbed water molecule decreases, the water molecules de-attached fast from the surface of the sensor.

### Sensing mechanism

**Figure 17:**
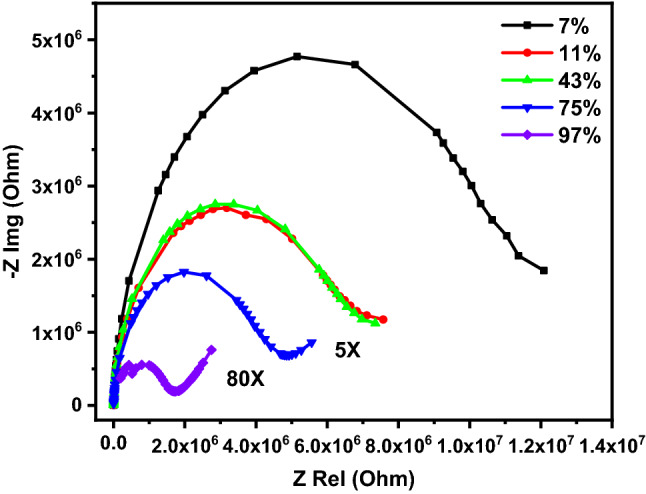
The Nequest plot of GQDs at different humidity levels from 50 up to 5 MHz.

The humidity sensing mechanism was studied using complex impedance spectroscopy (CIS). The real part (Z_Rel_) and imaginary part (− Z_Img_) of the impedance were measured at different humidity levels. The examined sensors were stabilized for 15 min at specific humidity level before acquiring the desired results. The sensors were measured in a frequency range from 50 Hz to 5 MHz at room temperature. The relationship between Z_Rel_ on X-axis and − Z_Img_ on Y-axis for GQDS and GQDs\g-C3N4 at different humidity levels is depicted in Fig. 17. The Nyquist plot for GQDs demonstrates a semicircle at low humidity level. A small tail starts to appear as the humidity level increases beyond 75%. More importantly, the curvature of the developed semicircle increases as the humidity increases. The sensing mechanism can be explained based on the Grotthuss chain reaction^41,61,62^. At low humidity level the adsorbed water molecules are dissociated on the surface of the sensor to form protons (H^+^) and hydroxyl groups (OH^−^). The abundance of hydroxyl group attached to the GQDs is the key factor responsible for generating hydronium ions. The water molecules are adsorbed sequentially to form multiple layers over sensing material. Despite other reported humidity materials, at low humidity level the water molecules are adsorbed physically and interacted with the surface hydroxyl group to generate hydronium ions. As the humidity increases (mid-level of humidity) the generated hydronium ions interact with the subsequent adsorbed water molecules, thereby the generated protons are mobile and can move freely between adjacent water molecules. The full mechanism can be described by the following equations:

7 $${\text{H}}_{{2}} {\text{O }} + {\text{ OH}}^{ - } \to {\text{H}}^{ + } + {\text{ 2OH}}^{ - }$$

8 $${\text{H}}_{{2}} {\text{O }} + {\text{OH}}^{ - } \leftrightarrow {\text{H}}_{{3}} {\text{O}}^{ + } + {\text{ OH}}^{ - }$$

9 $${\text{H}}_{{2}} {\text{O }} + {\text{ H}}^{ + } \leftrightarrow {\text{H}}_{{3}} {\text{O}}^{ + }$$

The humidity sensing behavior of the synthesized composite was compared with those obtained data from other researchers as tabulated in Table 5. The obtained results exhibited a wide span of working range, and low response and recovery time.

**Table 5:** performance of the proposed sensor in comparison with other reported sensors in previous works.

Sensing material	Fabrication method	Measurement range	Sensor Response Time (s)	Sensor Recovery Time (s)	Refs.
g-C3N4/GQDs nano composites	Hydrothermal Method	7–97% RH	44	10	This paper
MoS_2_/GOQD	Drop coating	11–97% RH	20	12	^63^
Flexible graphene quantum dots	Hydrothermal method	1–100% RH	12	43	^64^
N-S co-doped graphene quantum dots prepared from waste	Hydrothermal method	10–90% RH	15	55	^22^
CNT/GQD and CNT/PEDOT:PSS	Spin coating technique	20–80% RH	20	70	^65^

## Conclusion

In the present study, the ability of the developed g-C3N4/GQDs nano composite to be used as a humidity sensor was evaluated through some techniques like XRD, HR-TEM, FTIR, UV–Vis, Raman, XPS and BET surface area in addition to model molecule.

The following results are obtained:

- DFT:B3LYP/LANL2DZ model indicated that g-C3N4 increased the reactivity of GQDs. The g-C3N4/GQDs composite Show the ability to coordinate water molecules throughout Van der Waal interaction through the hydrogen of the carboxyl group at the surface of GQDs. This in turn leads to possible applications of carbon nitride/GQDs as humidity sensor.
- An average particle size for GQDs was estimated from XRD to be 5 nm.
- Well dispersed spherical particles on the surface of the 2D wrinkled overlapped graphene sheets was observed from HRTEM, also with average dimension of GQDs was about 4 nm which is compatible with that estimated from XRD pattern.
- In addition as calculated from Tuinstra and Koenig (TK) equation the average size is from 2 to 5 nm.
- The estimated BET surface area was 216, 313.4, and 545.4 m^2^/g for GQDs, g-C3N4, and GQDs\g-C3N4 respectively, the highest value was for GQDs\g-C3N4 may be attributed to the incorporation of GQDs between g-C3N4 sheets that preventing the agglomeration.
- From the humidity measurements, the g-C3N4 show no response to the variation in humidity so, it cannot be considered as a humidity sensor, while GQDs exhibits a distinct behavior compared to g-C3N4 when subjected to different levels of humidity. Also, the humidity response of GQDs/g-C3N4 composite at 100 Hz demonstrates a linear dependance of impedance as humidity increases.
- The response time for GQDs and GQDs/g-C3N4 was 27 s and 44 s respectively. While, the recovery times were estimated to be 80 s and 10 s respectively.
- Finally, based on the abovementioned results, the GQDs/g-C3N4 composite can be efficiently used as humidity sensor.

## Data Availability

All data generated or analyzed during this study are included in this submitted manuscript.
